# Supplementary material for: Factors associated with unmet healthcare needs in patients using Primary Care Access Points for unattached patients in Quebec (Canada)
Source: PLoS One. 2026 Jan 30;21(1):e0342106. doi: 10.1371/journal.pone.0342106 (PMC12857950; doi:10.1371/journal.pone.0342106)
Supplement: Attachment — Submitted filename: Response reviewers_Unmetneeds_GAP_2oct2025.docx [file pone.0342106.s001.docx]

Submission to PLOS One

Reviewers’ comments received September, 11^th^ 2025

Manuscript Title: “Factors associated with unmet healthcare needs in patients using Primary Care Access Points for unattached patients in Quebec (Canada)”

Date: October, 2^nd^ 2025

Dear Dr. Faisal Shaheen,

We thank the two reviewers and editor you their thoughtful and constructive feedback. We have carefully revised the manuscript to address all comments. We believe these revisions have strengthened the paper considerably. Below, we provide a detailed point-by-point response. All changes in the manuscript are highlighted using tracked changes.

Please note that after discussion with our statistician, the vulnerability index was revised to only consider participants who had not missing data on the four variables that constitute the index. The multivariable regression models were redone and are presented in the paper. Overall, only small changes were observed.

| **Reviewer 1 comments** |  |
| --- | --- |
|  | **Modification** |
| Strengths of the Study  - Use of a very large, multi-site patient sample enhances generalizability of findings.  - Patient-centered outcomes are measured using validated PREM tools, directly reflecting patient experiences.  - Application of the Andersen behavioral model allows nuanced analysis of predisposing, enabling, and need factors.  - Stratified regression models by GAP orientation provide granular insights into modality-specific issues.  - The authors contextualize findings within ongoing healthcare reforms and recent changes to physician incentive structures, revealing important external influences. | The authors would like to thank the reviewer for taking the time to review our article and provide valuable feedback. |
| #1  - Response Rate and Representativeness  The response rate in this study warrants critical attention in evaluating the generalizability of its findings. As recruitment relied on email invitations, there is a risk of underrepresenting vulnerable groups (elderly, low-income, or digital literacy-limited populations). Please provide further discussion of this limitation, and—if feasible—consider applying or discussing sample weighting strategies to address non-response and improve credibility. According to the manuscript: A total of 212,546 patients with valid email addresses were invited to participate in the survey from a pool of approximately 279,000 unattached patients registered across three local health territories (LHTs) in Quebec. Of these, 41,384 individuals responded, yielding an overall response rate of approximately 19% among those invited. Among respondents, 20,282 individuals both responded on their own behalf and had used the GAP service—forming the analytic sample. This implies that the analyzed data represents only about 9.5% of invited individuals, and approximately 7.3% of the total unattached patient population in the target regions. | The reviewer is correct. Our data collection was limited to patients with a valid email address, which indeed introduce a potential risk of underrepresenting vulnerable groups. We have acknowledged this limitation explicitly in the revised manuscript.  For their comment regarding the representativeness of the study population, unfortunately, we don’t have the characteristics of the population of unattached patients registered on the waiting list in the 3 LHTs and this population is changing daily with new patients being registered and other patients being removed from the list because they were assigned to a GP. For these reasons, weighting strategies to address potential bias were not feasible. To ensure this limitation was adequately addressed, we consulted with the senior statistician on our team. In the revised manuscript, we have expanded the Limitations section to clarify this point and to suggest data collection strategies for future studies that could better reach vulnerable groups not captured in our study. The following statement was added to the limitation section:  « The study sample was also limited to people with an email address, which likely led to under-estimation of the experiences of vulnerable individuals (i.e. older adults, individuals living in poverty or with low digital literacy). To reach these groups, additional strategies should be considered in future studies, such as postal questionnaires and phone administration [72, 73]. However, these data collection methods are typically more expensive. Further, the characteristics of the population of unattached patients registered on the waiting list in the 3 LHTs understudy are unknown, and this population is changing daily with new patients being registered, while others are removed after being assigned a GP. This limited our ability to compare the characteristics of the available sample and the target population and apply sample weighting to address the potential bias. » (p.23-24) |
| #2  - Limitations of the GAP Model for Core Primary Care Functions  The current GAP model is optimized for prompt, acute care. I recommend a more explicit discussion of the structural gaps in preventive, chronic, and continuous care for unattached patients, and—if possible—suggest strategies or policy directions to address these limitations. | Discussion section has been modified and restructured to improve clarity and depth. Specifically, we expanded the discussion of the gaps in preventive, chronic, and continuous care, and we further elaborated on the recommended policy directions to address these limitations. These revisions can be found on pages 21–22 of the manuscript. |
| #3.  - Definition of Unmet Need  As the measurement of unmet need was specifically focused on the GAP encounter for the main reported reason, emphasize this scope in both Results and Discussion. Also, mention as a limitation that multiple, concurrent unmet needs may exist, particularly among complex patients. | Thank you for pointing that out. We emphasized this in the result and discussion sections.  The following limitation was also presented in the limitation section:  « Unmet needs were only measured for the GAP service received for the main reason for call, while not considering the other health needs discussed during the call. This limitation could be even greater for complex cases and multimorbidity patients. » (p.24) |
| #4  - Patient–Provider Education in Team-Based Care  Since unmet need was substantially higher for non-physician provider orientations, the discussion should address not only patient expectations but also opportunities for better communication, patient education, and acceptance of diverse provider roles within interprofessional primary care. | Thank you for the comment. The following phrases were added to the discussion : « From an interprofessional perspective, a cultural shift is also needed within the population by improving communication with patients and enhancing their understanding of the scope and role of each professional. The Quebec ministry of health and social services’s main message ‘The right service, by the right person, at the right time!’ [66] should also be further promoted, for example through flyers in waiting rooms, social media, television and radio campaigns, as well as direct discussion with patients during consultations. » (pages 20) |
| #5  - Conclusion and Recommendation -  While the study offers valuable insights into the characteristics and experiences of GAP users who responded to the survey, its findings should not be generalized to all unattached patients in Quebec without caution. Future studies would benefit from: Comparative analyses of respondent vs. nonrespondent characteristics, application of population weights or statistical correction methods, and use of alternative survey modalities (e.g., phone, mail) to reach digitally underserved populations.  Overall, this is an important study that substantially contributes to our understanding of access models for unattached patients and the design of team-based primary care systems. The large-scale, real-world data, careful methodology, and clear policy relevance are strengths.  Provided that the authors address the above points—particularly around issues of representativeness, the definition of unmet need, and strategies to build acceptance and effectiveness of team-based care—I find this manuscript suitable for publication in PLOS ONE. | We sincerely thank the reviewer for their valuable comments, which have greatly enriched the manuscript. All of the points raised have been addressed. In particular, we clarified the issue of representativeness, clarified the definition of unmet need, and expanded the discussion on strategies to strengthen primary team-based care. |

| **Reviewer 2 comments** | |
| --- | --- |
|  | **Modification** |
| Dear Authors,  It is very interesting and valuable topic of research as the objectives of this study are to" 1) document the factors associated with unmet healthcare needs after receiving a GAP service and 2) assess if these factors vary according to GAP service received" So goals of research confirm the importance of the research as well as they are clearly specified. | We would like to thank the reviewer for providing valuable comments that improve the manuscript. |
| #1  Introduction contains not only the description of purposes of the research but also the importance of primary health and unmet needs are well explained. Also factors, which are responsible for unmet needs are mentioned and they apply to Canada. So, it would be worth to make also some literature research of healthcare system, which are similar type of Canada in purpose to see also what are the factors of unmet needs and then to consider them also if they are different. So, readers should be aware if they are the same etc.. | We conducted a literature research of relevant articles in universal healthcare systems. The following sentence was added in the introduction: « The factors documented in other countries with a universal healthcare system are similar, but the status of employment (unemployed) is frequently associated with unmet healthcare needs [29-31], whereas results were mixed in Canadian studies. » (p.2) |
| #2  Methodology is well explained and especially the questionnaire.  Results and discussion are well presented. However the practical and theoretical implications should be expended. | We thank the reviewer for this helpful comment. The practical and theoretical implications of our findings have been further developed and are now discussed in the opening paragraph of the Discussion section, as shown below:  « GAPs were implemented to help unattached patients navigate and access primary care services outside the emergency room, in response to increasingly long wait times, while waiting to be attached through the centralized waiting lists. To our knowledge, this study is the first to document the factors associated with perceived unmet healthcare needs after receiving GAP service for the main reason for call and assess whether those factors vary according to GAP orientation. Using the Andersen framework [52], the study contributes to the limited literature on unmet healthcare needs among unattached patients. This is also the first study to evaluate GAP orientations from the patient perspective. Given that the GAP was implemented province-wide and is available to more than 1.6 million inhabitants, identifying the characteristics of unattached patients who are at higher risk of reporting unmet needs is fundamental to assess whether GAP’s joint strategy–ensuring appropriateness of healthcare resources and optimal use of the expertise of health professionals other than family physicians–meets patient needs. Our study will also provide insights into GAP orientations that may be further developed or improved to meet more effectively the needs of unattached patients. » (p.17-18) |
