# Supplementary material for: Factors associated with unmet healthcare needs in patients using Primary Care Access Points for unattached patients in Quebec (Canada)
Source: PLoS One. 2026 Jan 30;21(1):e0342106. doi: 10.1371/journal.pone.0342106 (PMC12857950; doi:10.1371/journal.pone.0342106)
Supplement: Attachment — Submitted filename: Response reviewers_Unmetneeds_GAP_11dec2025.docx [file pone.0342106.s002.docx]

Submission to PLOS One

Reviewers’ comments received November, 25^th^ 2025

Manuscript Title: “Factors associated with unmet healthcare needs in patients using Primary Care Access Points for unattached patients in Quebec (Canada)”

Date: December 11^th^, 2025

Dear Dr. Faisal Shaheen,

We thank the two reviewers and editor you their thoughtful and constructive feedback. We have carefully revised the manuscript to address all comments made by the reviewer. We believe these revisions have strengthened the paper considerably. Below, we provide a detailed point-by-point response. All changes in the manuscript are highlighted using tracked changes.

| **Reviewer 1 comments** |  |
| --- | --- |
|  | **Modification** |
| Thank you for your detailed response addressing the concerns raised regarding the response rate and representativeness. I appreciate the authors’ clear acknowledgment of the limitations inherent in using an email-based recruitment strategy, as well as the expanded discussion provided in the revised manuscript. Your explanation regarding the lack of available population characteristics for unattached patients across the three LHTs—and the high turnover within the CWL—offers a reasonable justification for why weighting strategies were not feasible.  That said, I encourage the authors to further strengthen the manuscript in two areas:  1. Interpretation of the potential impact on study findings.  While the limitations are acknowledged, the revised version does not sufficiently discuss how low representativeness may affect the direction or magnitude of key results. Because the analytic sample represents only about 7% of the unattached population, it would be helpful to explicitly address how overrepresentation of individuals with higher digital access might influence estimates of unmet healthcare needs or the distribution of GAP orientations. | We would like to thank the reviewer for taking the time to review our article and provide valuable feedback.  We added further explanation to address the issue of low representativeness and its impact on the results:  « The study sample, representing only 7% of all unattached patients registered on the centralized waiting list in Montérégie, was limited to people with an email address, which may have led to the overrepresentation of individuals with high digital literacy. Unattached patients who are not registered on the centralized waiting list were also not captured in our study. Therefore, vulnerable individuals (i.e. older adults, individuals living in poverty or with low digital literacy) may be underrepresented in the study sample. » (p.21-22) |
| 2. Consideration of alternative post-hoc adjustment approaches.  Even if full weighting was not possible, it may still be beneficial to briefly note whether any partial adjustment strategies were considered—such as LHT-level calibration, or comparisons with publicly available demographic data. Stating that such options were evaluated but deemed infeasible would improve transparency in methodological decision-making.  Your revisions are appreciated and address the core of the concern; however, a modest additional elaboration on these points would better contextualize the implications of non-response and representativeness limitations for readers and policymakers who may rely on these findings. | Unfortunately, data on unattached patients at the regional level are not available. As a result, calibration at the LHT level or comparisons with publicly available demographic data are not possible. This information has been added to the limitations as follow:  « Further, data on the characteristics of unattached patients at the regional level are not available, and this population is changing daily with new patients being registered, while others are removed after being assigned a GP. » (p. 22)  However, we compared the characteristics of the population of Montérégie (attached and unattached patients) and with those of the province of Quebec using publicly available demographic data. The following statement was added to the discussion:  « However, public data available for the entire population of Montérégie (attached and unattached individuals) showed that the residents are more economically advantaged (higher employment rate, average wage and disposable income/capita) and the region has more immigration compared to the province of Quebec [73]. » (p.22) |
| Figures and Tables  Please improve the readability of Figure 2 by using clearer labels or considering an alternative graphical format.  Ensure consistent formatting of statistically significant results across tables (e.g., bolding, decimal places).  Add the following footnote to Table 1 to define all abbreviations:  LHT: Local Health Territory  CWL: Centralized Waiting List for Unattached Patients  GAP: Primary Care Access Point for Unattached Patients | Tables  The tables were revised to ensure that all significant results are in bold and results are presented with two decimals. A Footnote was added to Table 1 as suggested.  Figure 2  We made the labels darker and increased their font size. The resolution of the figure was also enhanced. |
| 2. Clarity and Grammar  Minor edits are needed to improve clarity:  “an in-depth exploration the reasons” → “an in-depth exploration of the reasons” | Thank you for pointing that out. The error was corrected. |
