# Supplementary material for: Factors associated with unmet healthcare needs in patients using Primary Care Access Points for unattached patients in Quebec (Canada)
Source: PLoS One. 2026 Jan 30;21(1):e0342106. doi: 10.1371/journal.pone.0342106 (PMC12857950; doi:10.1371/journal.pone.0342106)
Supplement: Attachment — Submitted filename: Response reviewers_Unmetneeds_GAP_08jan2026.docx [file pone.0342106.s003.docx]

Submission to PLOS One

Reviewers’ comments received January, 5^th^ 2026

Manuscript Title: “Factors associated with unmet healthcare needs in patients using Primary Care Access Points for unattached patients in Quebec (Canada)”

Date: January 8^th^, 2026

Dear Dr. Faisal Shaheen,

We thank the reviewers for their thoughtful and constructive feedback. We have carefully revised the manuscript to address all comments made by the reviewer. We believe these revisions have strengthened the paper. Below, we provide a detailed point-by-point response. All changes in the manuscript are highlighted using tracked changes.

| **Reviewer 1 comments** |  |
| --- | --- |
|  | **Modification** |
| The revised manuscript has improved substantially since the previous submission and now presents a clear and well-structured analysis of unmet healthcare needs among unattached patients using Primary Care Access Points (GAP). The study addresses a relevant primary care issue, and the analytical approach is generally appropriate. Most of the major concerns raised in the previous review have been adequately addressed.  However, a few minor issues related to reporting clarity remain and should be corrected before final acceptance. | The authors would like to thank the reviewer for taking the time to review our article and provide valuable feedback. |
| First, in Table 3, adjusted odds ratios (AORs) are presented, but the table footnote does not specify which covariates were included in the adjusted model. For transparency and ease of interpretation, the authors should clearly state in the footnote the variables adjusted for in the multivariable analysis. | Thank you for pointing that out. Table 3 was revised and the following footnote was added: « Multivariable models were adjusted for all covariables included in Table 3. » (p.13) |
| Second, Table 4 raises the same issue as Table 3. Adjusted estimates are shown, yet the corresponding footnote does not describe the adjustment variables. Consistent reporting across tables is important, and Table 4 should include a footnote specifying the covariates used in the adjusted analysis, in parallel with Table 3. | Table 4 was revised and the following footnote was added: « Multivariable models were adjusted for all covariables included in Table 4 with the exception of emergency room model, which was adjusted for all covariables except frequency of emergency room visits. » (p.15) |
| Third, all tables should be self-explanatory, with clear definitions of abbreviations used. In particular, the abbreviation “GAP” should be defined in the footnote of each table where it appears, even if it has been defined elsewhere in the text. | Tables 2, 3 and 4 were revised and the following footnote was added: « GAP: Primary Care Access Point for Unattached Patients (Guichet d’accès à la première ligne); LHT: Local Health Territory ». |
